# Recurrence Methods for the Identification of Morphogenetic Patterns

**DOI:** 10.1371/journal.pone.0073686

**Published:** 2013-09-16

**Authors:** Angelo Facchini, Chiara Mocenni

**Affiliations:** 1 Enel Foundation, Rome, Italy; 2 Department of Information Engineering and Mathematical Sciences, University of Siena, Siena, Italy; University of Calgary, Canada

## Abstract

This paper addresses the problem of identifying the parameters involved in the formation of spatial patterns in nonlinear two dimensional systems. To this aim, we perform numerical experiments on a prototypical model generating morphogenetic Turing patterns, by changing both the spatial frequency and shape of the patterns. The features of the patterns and their relationship with the model parameters are characterized by means of the Generalized Recurrence Quantification measures. We show that the recurrence measures Determinism and Recurrence Entropy, as well as the distribution of the line lengths, allow for a full characterization of the patterns in terms of power law decay with respect to the parameters involved in the determination of their spatial frequency and shape. A comparison with the standard two dimensional Fourier transform is performed and the results show a better performance of the recurrence indicators in identifying a reliable connection with the spatial frequency of the patterns. Finally, in order to evaluate the robustness of the estimation of the power low decay, extensive simulations have been performed by adding different levels of noise to the patterns.

## Introduction

Morphogenesis is the mechanism for which spatial structures and patterns form spontaneously in biological and biochemical systems. This phenomenon can be explained by assuming that at least two species, an activator and an inhibitor, are interacting in a spatial domain subject to reaction and diffusion processes of different intensities [1]. The striking theory developed by Alan Turing provided a reliable framework for the physical and mathematical understanding and modeling of such complex systems. Although this mechanism is well known and measurable in biology and medicine at different scales, such as molecular and cellular, few studies handle the problem of matching mathematical and numerical models with real measurements. The analysis of these kind of complex spatio-temporal data is complicated by the presence of small disturbancies in the measurements [2], preventing successful application of segmentation techniques for identifying the typical elements in the structures. The presence of noise and quasi periodicities in the measurements introduces further elements of uncertainty and compromises the application of satisfactory analysis in the frequency domain.

**Figure 1:**
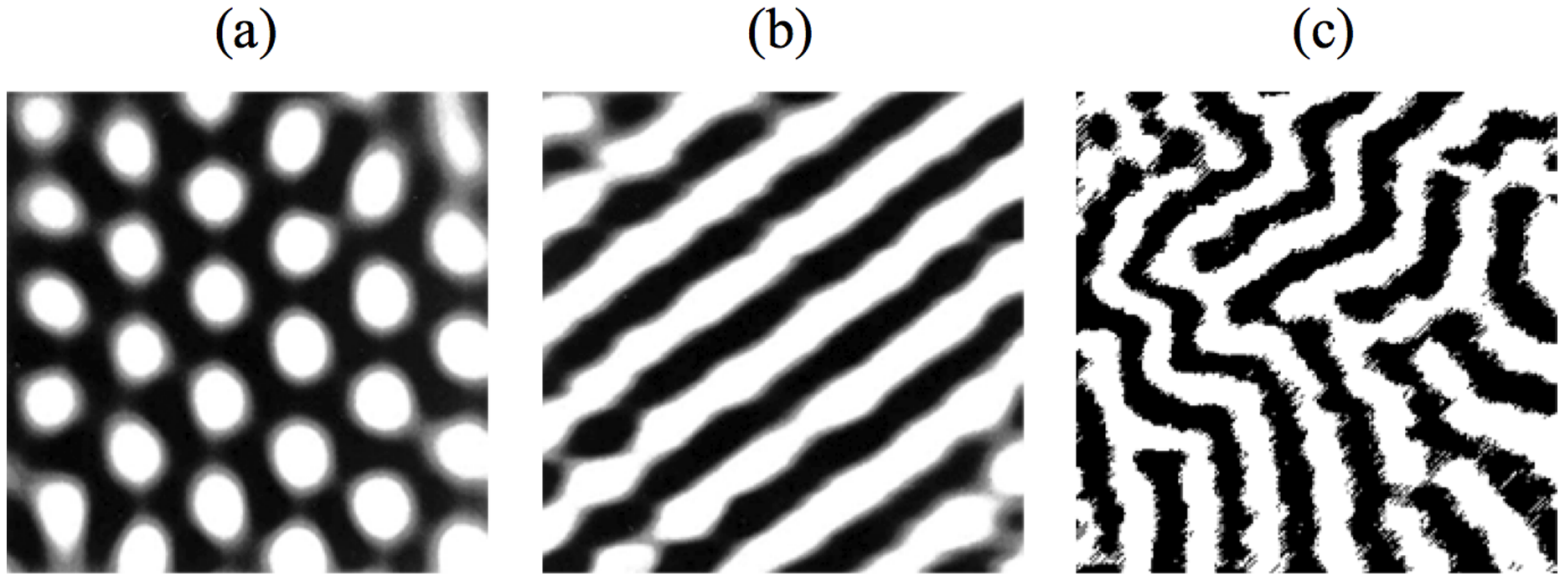
Different types of stationary Turing Structures: a) Hexagons b) Stripes c) Labyrinthine.

In order to find innovative methodologies for identifying and modelling patterned data arising from biological and biochemical reaction-diffusion processes, we observe that the main feature of such data is the presence of regular structures involving typical patterns (see, for example, spots and stripes of Figure 1. It is straightforward recognizing in these images the quasi periodicity in space, for which a typical element is almost regularly recurrent.

The idea of using methods based on repetitions and recurrences for studying experimental patterns can be related to the concept of recurrence for complex systems, initially developed by Poincaré [3]. Indeed, this concept was used by Poincaré in the field of dynamical systems to solve the three body problem, and by Kac [4] for discrete stochastic systems. For time series, the concept of recurrence was carried out by Eckmann, who introduced the Recurrence Plot (RP) as a visual tool designed to display recurring patterns and to investigate nonstationary patterns [5]. In the field of time series analysis, RPs found a wide range of applications to the analysis of nonstationary phenomena, such as biological systems, speech analysis, financial time series, and earth sciences (see [6] and literature cited therein). The popularity of RPs lies in the fact that their structure is visually appealing and allows for the investigation of high dimensional dynamics by looking at a simple two-dimensional plot. Furthermore, by means of the Recurrence Quantification Analysis (RQA) [7], the RP has been used as a tool for the exploration of bifurcation phenomena and changes in the dynamics when dealing with nonstationary and short time series [8].

The extension of the concept of recurrence for higher dimensional data, namely the Generalized Recurrence Plot (GRP) [9], provided interesting tools for the analysis of complex dynamics in spatially distributed systems, such as chlorophyll bloom in oceans, bacterial growth and chemical waves [10], [11]. Furthermore, GRPs and Generalized Recurrence Quantification Analysis (GRQA) have been successfully exploited in the identification of structural changes in complex spatially distributed systems, such as the Complex Ginzburg-Landau equation [12] and in the detection of different Turing bifurcations mechanism in the Belousov-Zabotinsky reaction performed in a oil-water microemulsion [13]. Because of the nonlinear features of the models leading to pattern formation, the problem of state space reconstruction and parameter identification is a hard task. State space reconstruction of a spatio-temporal dynamical system has been investigated in lattice dynamical systems [14], while a method for spatial forecasting from single snapshots has been proposed in [15]. Furthermore, in several cases, one has to cope with the problem of understanding the dynamics of a system by using only a limited number of observations.

This work addresses the problem of identifying a correlation between the structure of patterns and model parameters in a prototypical model showing Turing pattern formation. Two numerical experiments are performed by varying the parameters controlling the shape and spatial frequency of the patterns, respectively. Our aim is to establish a functional relationship between the recurrence indicators and the model parameters by means of GRPs and GRQA. We will show that the functional form of the GRQA measures strictly depends on one of the model parameters. The relationship can be used to identify suitable values of the parameters responsible for the generation of patterns, which can be a hard task in experiment design and practical applications. Moreover, this relationship is shown to be robust with respect to noise levels lower than 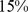.

## Results and Discussion

Two numerical experiments have been performed by varying the parameters 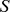 and 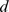 of the model described in the Materials and Methods section. The parameter 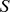 controls the frequency of the patterns generated, while by varying the diffusion coefficient, 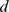, the patterns are formed or change from spot to labyrinthine. Therefore, by continuously varying these parameters we are able to obtain a set of stationary solutions showing the trajectories from higher to lower pattern frequencies and from spot to labyrinthine structures.

**Figure 2:**
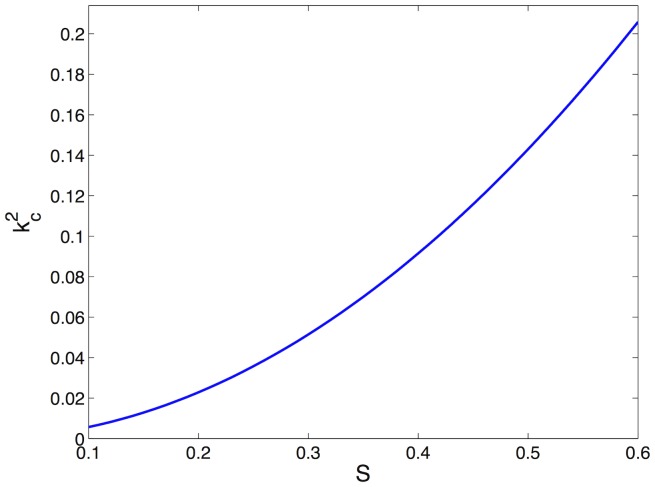
Critical wavenumbers 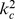 for increasing values of parameter *S*. All the other parameters are fixed: 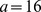, 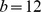, 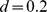.

In the first experiment, we set 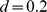, while the spatial frequency of the spot patterns is increased by varying the parameter 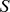 in the range 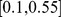. The critical wavenumber 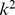, which accounts for the spatial frequency of patterns, with respect to parameter 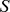 and for fixed 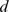 is shown in Figure 2.

In the second experiment, the diffusion coefficient 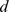 is varied in the range 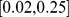, while 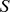 is kept constant (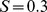). In this case the spatial frequency of the structures should not change significantly, while the appearance of the patterns ranges from labyrinthine structures (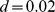) to spots (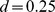).

In the following we report the evolution of the recurrence indicators 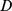 and 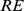, described in the Materials and Methods section, with respect to 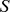 and 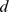. Furthermore, we will show how the distribution of the diagonal lines 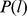 of the RP changes according to the characteristics of the pattern analyzed.

### Experiment 1

**Figure 3:**
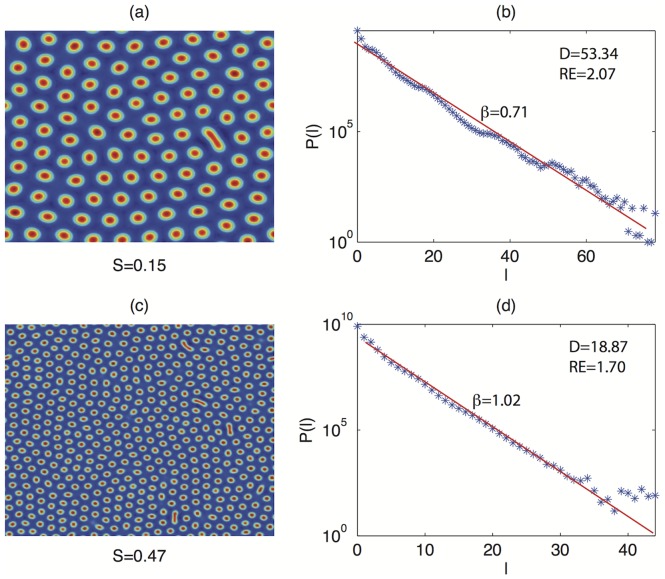
Spot patterns for different values of parameter 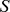 (panels (a), (c)) and corresponding histograms of line lengths 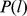 (panels (b), (d)). As 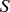 increases, the values of the exponential decay of 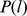 raise from 0.71 (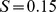) to 1.02 (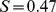).

Figure 3 shows the results of the analysis based on recurrences: panels (a) and (c) report the variable 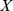 showing spot patterns for 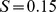 and 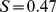, respectively. Panels (b) and (d) report the corresponding line lengths distributions 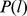. A first inspection of 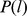 suggests that the line length decays exponentially (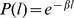) with different exponents: 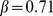 in the case of smaller spatial frequency (panel (b)) and 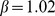 in the case of higher spatial frequency (panel (d)). In fact, as the frequency increases, the size and distance of the spots decreases, resulting in a smaller number of long diagonal lines. Notice that 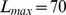 for 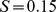 and 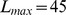 for 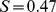, where 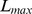 is the maximum line length of the RP.

The comparison of 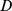 and 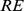 offers a further validation: 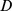 dramatically decreases from 53.34 to 18.87, while 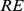 shows only small variations according to the findings (see [10]) that this indicator is more sensitive to changes in the small scale structure of patterns, such as the shape of single patterns, which does not change when increasing the spatial frequency. Furthermore, the strong decrease of 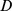 with 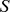 suggests a deeper investigation of the relationship of the recurrence indicators from 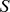.

We then performed an additional experiment by continuously varying 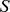 in the interval 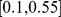. The results are reported in Movie S1, which is organized as follows: for each 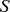, panel (a) shows the spot patterns; panel (b) reports the values of 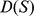 with respect to 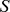; panel (c) reports the computation of the power spectral density of each image showed in panel (a), and, finally, panel (d) reports the values of the main frequency 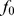 of the patterns (the main frequency has been computed by smoothing the power spectrum in the 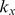 direction and by extracting its maximum value).

**Figure 4:**
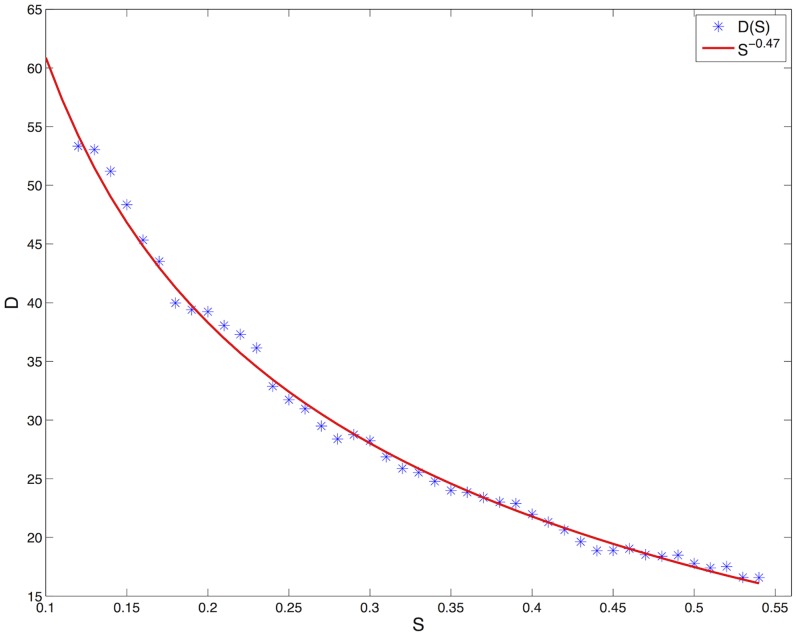
Decreasing evolution of the recurrence indicator 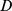 for increasing 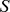, which is the parameter correlated to the spatial frequency of the patterns. 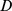
 decreases with the power law 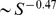.

**Figure 5:**
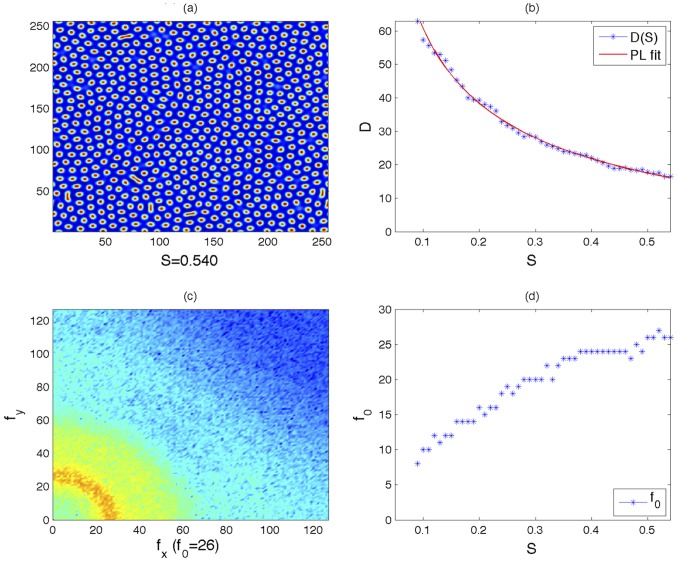
Snapshot of the last frame of Movie S1. Panel (a): spot distribution for 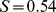. Panel (b): evolution of 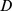 for increasing 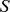 according the power law decay discussed in the Results and Discussion section. Panel (c): 2D-FFT showing the power spectral density of the pattern. Panel (d): evolution of the peak 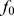.

Figure 4 shows with greater detail that as 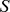 is increased, 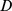 decays according the power law 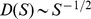. This result clearly indicates the existence of a functional relationship between the determinism 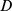 and the parameter 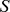, which controls the spatial frequency of the patterns in the model. The same relationship is hardly obtained by computing the power spectral density of the image. Indeed, as one can see in panels (c) and (d) of Figure 5 the power spectrum is noisy, and an accurate estimation of 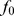 is difficult. Furthermore, 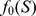 does not seem to evolve according to a quadratic function. In conclusion, the determinism represents a good measure for characterizing, both qualitatively and quantitatively, patterns arising from (potentially unknown) reaction-diffusion models.

### Experiment 2

**Figure 6:**
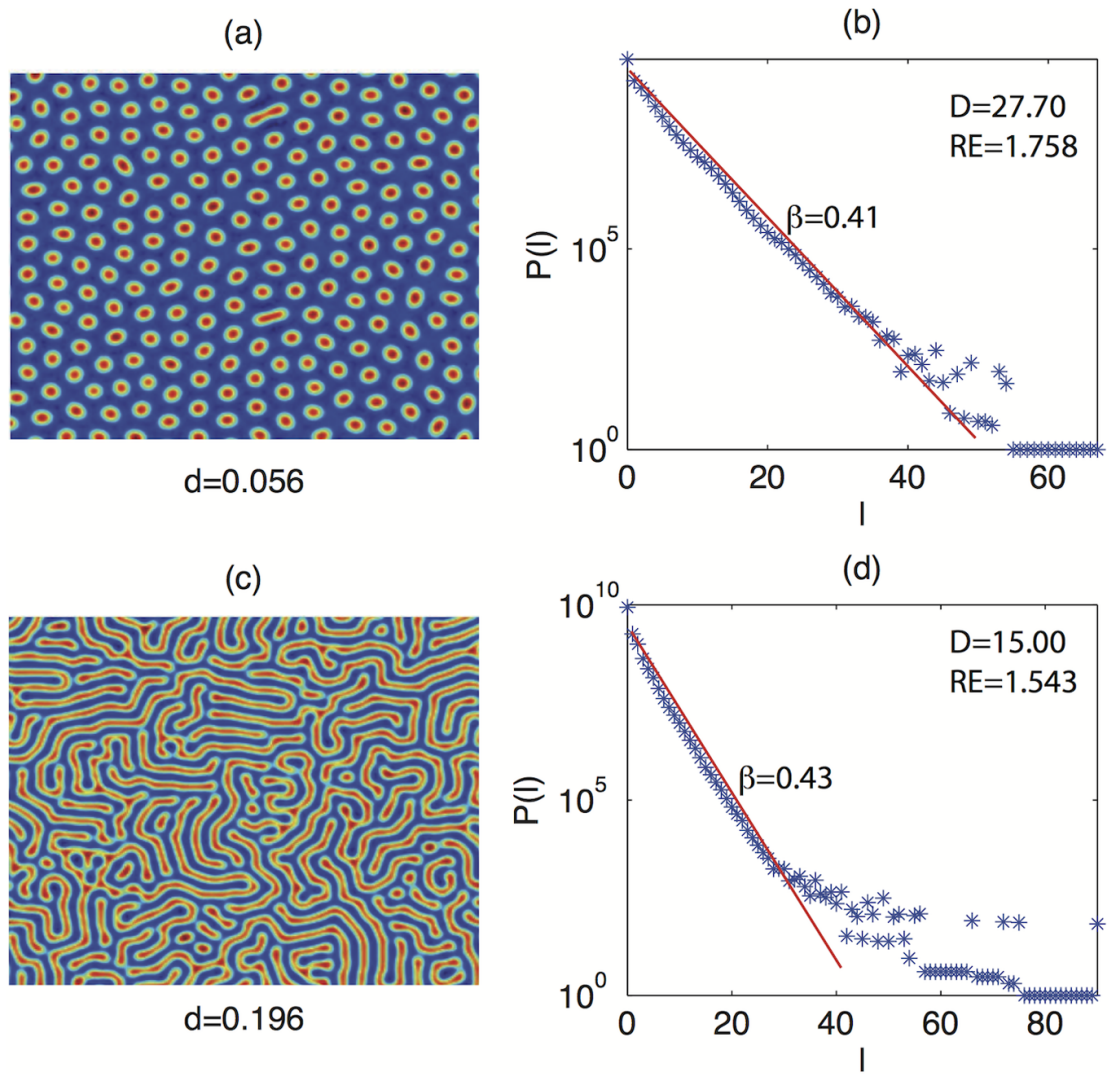
Spot patterns for *d*  =  0.056 (panel (a)) and labyrinthine structures for *d*  =  0.196 (panel (c)). Corresponding histograms of line lengths 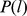 (panels (b) and (d)). By comparing panels (b) and (d) we notice that the values of the exponential decay and 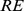 are similar, while the values of 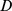 are considerably different.

In the second experiment we fix the spatial frequency of the patterns by maintaining 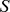 constant and by varying 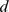 in the range 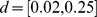. This produces patterns with the same spatial frequency, but of different typology. Panels (a) and (c) of Figure 6 show the patterns for 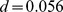 and 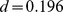, respectively, while panels (b) and (d) report the distribution of the line lengths 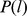. Analogously to the first experiment, we look at the distributions of the diagonal lines 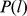. Under the new experimental conditions 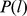 decays exponentially until the value 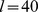 is reached, with similar decay exponents (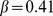 vs 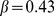). This is not surprising because the spatial frequency of the patterns is very similar, and the line lengths, at least for the first part of the distributions, are not affected by the global shape of the patterns. The same considerations hold for 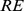. On the contrary, the value of 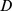 is strongly different: 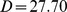 for the spots (panel (a)) and 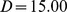 for the labyrinthine structures (panel (c)), reflecting the fact that, under the point of view of the global appearance of the pattern, the figure reported in panel (c) presents more complex structures, as demonstrated by the fat tail of 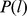 in panel (d). This experiment confirms that 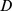 is a powerful measure for the characterization of spatial patterns generated by reaction diffusion models.

### Effect of Noise

The methods based on time recurrences have been shown to be robust with respect to noise and measurement errors [6], [16], providing suitable tools for analyzing data collected through real experiments.

**Figure 7:**
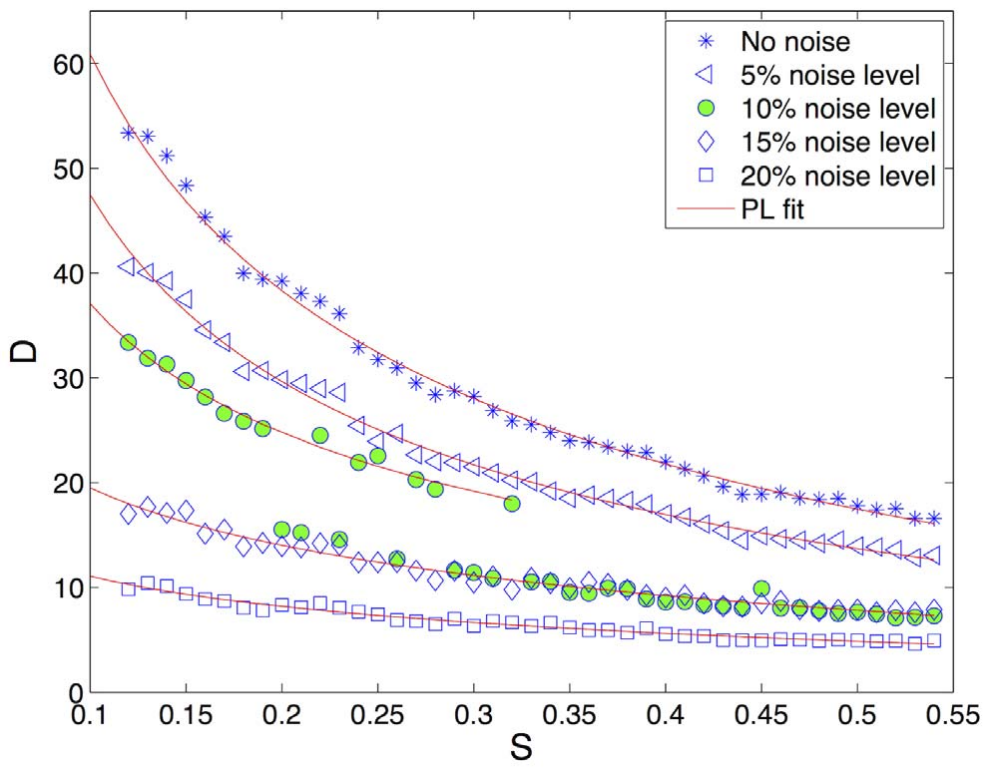
Determinism of the patterns for increasing values of parameter 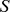 (see also Figure 4) and different noise levels. Specifically, Determinism is reported in the cases of no noise (stars), 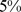 noise (triangles), 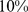 noise (filled green circles), 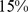 noise (diamonds) and 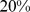 noise (squares).

In order to test and eventually find a similar robustness for methods based on spatial recurrences, the spot patterns analyzed in experiment 1 have been corrupted by noise with increasing intensity. Figure 7 reports the evolution of Determinism with respect to the parameter 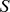 for increasing noise levels of 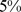, 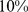, 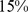 and 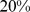, while the power law decays of 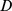, obtained by a fitting procedure, are reported in Table 1. The corresponding fitting curves are depicted in the figure by red lines. As the reader can see, the exponent 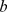 is consistent with the values obtained without noise and for noise levels of 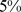 and 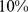. For noise levels of 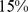 and 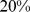, the fitting parameter changes significantly, showing that even if Determinism is still behaving according to a power law, the estimation of the decay exponent 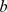 is less reasonable.

**Table 1:** Fitting parameters of the power low 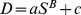 between Determinism and 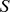 for different values of noise: 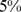, 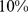, 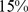 and 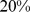.

	a	b	c	*R* ^2^	RMSE
no noise	29.79	−0.4508	−23.24	0.995	0.78
5% noise level	20.08	−0.4891	−14.48	0.993	0.72
10% noise level	16.69	−0.4441	−9.336	0.982	0.66
15% noise level	28.47	−0.1908	−24.68	0.972	0.51
20% noise level	18.03	−0.1666	−15.38	0.968	0.29

The last two columns report the 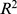 and the Residuals Mean Square Error (RMSE), respectively.

Moreover, it is worth noticing that between noise levels of 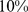 and 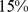, there exists a threshold separating reasonable and unsatisfactory estimations of the power law relationship. Indeed, the first part of the curve (green circles in Figure 7), corresponding to large size patterns, shows a satisfactory value for 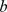 (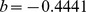), while intermediate-sized patterns, obtained with 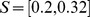, show an interesting intermittency between two different behaviors. Finally, for values of 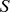 greater than 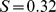 (small-sized patterns), the decay falls very close to the one corresponding to 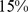 noise (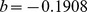). As a possible explanation for this behavior we could consider that as the patterns become smaller and smaller (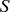 increasing), the artifacts introduced by noise can deeply modify the structure of the spots or even disrupt them. This fact is also confirmed by the results reported in [10], where it has been shown that Determinism of spatio-temporal systems decreases in a nonlinear and accelerating way with respect to the increase of noise.

### Conclusions

This paper addressed the problem of analyzing morphogenetic patterns emerging from nonlinear systems through physical mechanisms leading to Turing instabilities. The analysis is performed by using a set of recurrence indicators and, in particular, by means of the Generalized Recurrence Plots and Generalized Recurrence Quantification Analysis.

The results clearly show that the method carries out important insights about the structure of patterns and the evolution of such structures under different parametric conditions. Specifically, the main result concerns with the clear identification of the mathematical relationship between the parameter 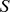, related to the spatial frequency of the patterns, and the recurrence indicator 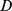, which accounts for the global appearance of the patterns.

Furthermore, our results will help the experimental scientist in identifying unknown parameters by using the information provided by the recurrence measures. Furthermore, when dealing with experiments where a strong sensitivity to the spatial frequency is present, the proposed method exploits the spatial recurrence properties for retrieving reliable information about the patterns. The obtained results have also been validated by adding noise to the patterns. In this case we found that the power low relationship between the Determinism and the parameter 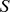 can be appropriately estimated even for noise levels lower than 15%.

Future developments will be devoted to the application of the proposed methodology for the parametric identification of experimental systems, such as bacterial growth and reaction diffusion systems performed in micro-emulsions.

## Materials and Methods

### The Turing Instability

In 1952 Turing [1] developed the original idea that coupling between reactions and diffusion of chemical species might play a role in morphogenesis, i.e. in the creation of differentiated structures in living organisms out of initially identical elementary cells. Turing showed that a uniform state may, in some circumstances, evolve because of a diffusive instability towards a new state in which the concentrations originate stationary structures organized in space. This spontaneous pattern-forming instability can occur only in systems maintained out of the spatial equilibrium and in which auto-activation processes are present. Therefore, a spatial pattern settles down because of a balance between the local activation processes and the long-range inhibition provided by molecular diffusion. This mechanism is quite general and hence the principle of a Turing instability can be recovered in other fields, such as heterogeneous catalysis, nonlinear optics, gas discharges, semiconductor devices, and materials irradiated by energetic particles or light. The common denominator of these phenomena is that they can be modeled by reaction-diffusion equations, such as those that naturally describe chemical systems. In all cases, the wavelength of the Turing-type spatial patterns accounts for the balance between the reaction-type mechanisms and the diffusion-like transport processes and is, therefore, intrinsic to the system. Figure 1 shows some of the stationary patterns (also known as Turing Structures, TS) generated by a reaction diffusion system; the type and the shape of TS depend on the values of the model parameters and on the boundary conditions.

### Recurrence Based Methods

In this section we provide only basic notions on recurrence methods for spatial data (for a deeper treatment on time series analysis the reader is referred to [6]).

In the case of a *d*-dimensional data-set, the Recurrence Plot is defined, according to [9], by:
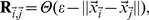
(1)where 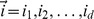 is the *d*-dimensional coordinate vector and 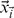 is the associated phase-space vector. This RP, called *Generalized Recurrence Plot (GRP)*, accounts for recurrences between the *d*-dimensional state vectors and presents a linear manifold of dimension 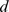 for which 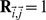, 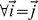, called the hypersurface of Identity (HOI).

We now consider spatially distributed systems at a certain (fixed) time of their evolution. In the particular case of 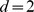, the single variable discretized solution of a two dimensional system can be visualized as an image, i.e. a two-dimensional object composed of scalar values, for which the GRP reads:
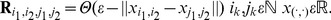
(2)


Each black dot in the GRP represents a spatial recurrence between two pixels, and every pixel is identified by its coordinates 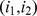, being 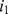 and 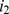 the row and the column index, respectively. In this case, the recurrence plot is four-dimensional and the HOI is generalized by a two-dimensional identity plane, defined by setting 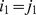 and 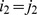.

A visual inspection of the four dimensional RP is possible only by projections in three or two dimensions. Although this is possible (see e.g [9], page 548), relevant information is hard to extract, and one must cope with the fact that GRPs lose their visual appeal. Despite this drawback, RQA can be easily generalized to GRQA by considering that the quantification is performed on the basis of the diagonal line lengths distribution 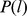. Then, providing a new definition of diagonal line, the quantification indicators are the same for both temporal and spatial case.

In the GRP the diagonal lines find an equivalent in diagonal patches of length 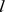, defined as follows (details are given in [9]):
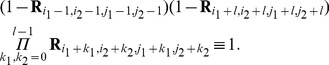
(3)


In [10] a different definition of structure of length 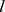 was given: instead of looking for two-dimensional patches, we look for the distribution of line segments in the four dimensional GRP. This is done by sampling the patch structures with lines. This can be obtained by looking for the recurrences only in the diagonal direction (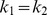). With this assumption, the formulation of diagonal line reads:
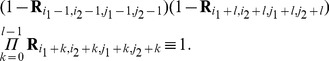
(4)


Focusing on isolated points and lines parallel to the HOI, the recurrence indicators can be generalized and the most important of which are *Recurrence Rate* 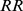, *Determinism* 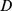, and *Recurrence Entropy* 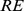.

The 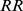 is defined as:
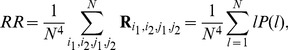
(5)and represents the fraction of recurrent points with respect to the total number of possible recurrences. It is a density measure of the RP.

The Determinism, defined as:
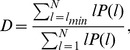
(6)is the fraction of recurrent points forming diagonal structures with a minimum length 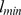 with respect to all recurrences. For choosing 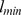 no theoretical guideline is provided and this choice is usually made by means of empirical considerations, such as by taking into account the average size of the patterns or the role of noise in the image. 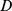 provides a measure of the global appearance of the patterns. For example, highly regular patterns, such as, e.g., periodic structures, will produce high values of 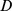 (more than 50%) since the recurrence points are mainly organized in diagonal lines. On the other side, random or poorly structured patterns are characterized by small values of 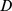 (0.5–1%).

The Recurrence Entropy, defined as:
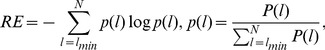
(7)is a complexity measure of the distribution of the diagonal lines in the RP.

It refers to the Shannon entropy with respect to the probability of finding a diagonal line of exactly length 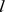. For periodic structures or uncorrelated noise the value is small (0.5–0.8), while for chaotic systems is higher (1.5–2.5). Under the point of view of the pattern, 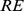 is related to the small scale structure of the image.

The computation of the measures based on the diagonal lines and their distribution provides valuable information about the structure of the RP. For the application of RQA to spatial systems the reader is referred to [9], [10].

### A Prototypical Model Generating Morphogenetic Patterns

In this paper we considered the model developed by Bard [17], designed to model and reproduce mammalian coat patterns. This model, although simple, describes a nonlinear reaction-diffusion kinetics for simulating Turing patterns. The model generates spots of different complexity, such as rings, and both vertical and horizontal stripes, as well as a variety of labyrinthine structures. The model equations are the following:
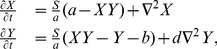
(8)where 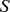 is a constant controlling the spacing of the patterns, 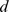 is the ration between the diffusion coefficients of the two species 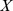 and 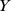, 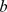 is the concentration of the enzyme in the domain, and 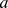 is a normalization constant. The presence of 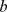 acts as a pattern-formation switch; if 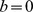, there is a normal stable equilibrium 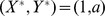; if 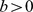 the equilibrium 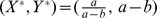 is unstable for 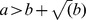.

In the last conditions, we observe the formation of spatial patterns whose spatial frequency and shape depend on the diffusion coefficient 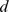 and on parameter 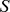.

The formation of the patterns is the result of the propagation of spatial unstable waves, whose wave number range 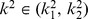 can be computed as a direct consequence of the instability conditions of the equilibrium point. The verification of the Turing instability conditions is straightforward, as described by Murray [18] (see section 2.3 of volume II).

To the purposes of this work, the model described in equation (8) has been simulated by taking 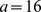 and 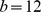 and varying 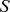 and 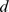. The numerical solutions 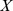 and 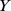 are then analyzed by means of the recurrence indicators Determinism and Recurrence Entropy.

## Supporting Information

Movie S1
**Spot distribution for 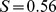 (panel (a)); Evolution of 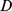 for increasing 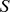 according the power law decay discussed in the Results and Discussion section (panel (b)); 2D-FFT showing the power spectral density of the pattern (panel (c)); Evolution of the peak 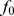 (panel (d)).**
(MP4)
